# Prevalence and factors associated with NAFLD detected by vibration controlled transient elastography among US adults: Results from NHANES 2017–2018

**DOI:** 10.1371/journal.pone.0252164

**Published:** 2021-06-03

**Authors:** Xiaotao Zhang, Natalia I. Heredia, Maya Balakrishnan, Aaron P. Thrift

**Affiliations:** 1 Department of Medicine, Section of Epidemiology and Population Sciences, Baylor College of Medicine, Houston, Texas, United States of America; 2 Department of Epidemiology, Division of Cancer Prevention and Population Sciences, The University of Texas MD Anderson Cancer Center, Houston, Texas, United States of America; 3 Department of Health Promotion & Behavioral Sciences, The University of Texas Health Science Center at Houston, School of Public Health, Houston, Texas, United States of America; 4 Department of Medicine, Section of Gastroenterology and Hepatology, Baylor College of Medicine, Houston, Texas, United States of America; 5 Dan L Duncan Comprehensive Cancer Center, Baylor College of Medicine, Houston, Texas, United States of America; McMaster University, CANADA

## Abstract

**Background:**

Non-alcoholic fatty liver disease (NAFLD) is increasingly common in the adult population. In the United States, the overall burden of NAFLD is unknown due to challenges with population-level NAFLD detection. The purpose of this study was to estimate prevalence of NAFLD and significant NAFLD fibrosis and identify factors associated with them in the U.S.

**Methods:**

Data came from the 2017–2018 cycle of National Health and Nutrition Examination Survey. We defined NAFLD by controlled attenuation parameter (CAP) scores of ≥248 dB/m in absence of excessive alcohol use and viral hepatitis. We defined significant fibrosis as Vibration controlled transient elastography (VCTE) liver stiffness measurements (LSM) value ≥7.9 kPa. We calculated the adjusted odds ratio (OR) and 95% confidential intervals (CI) for associations with NAFLD and significant NAFLD fibrosis using multivariable logistic regression.

**Results:**

Overall, among 4,024 individuals aged ≥20 years included in the analysis, 56.7% had NAFLD by CAP. In comparison, when defined by elevated liver enzymes, NAFLD prevalence was 12.4%. The prevalence of significant NAFLD fibrosis by VCTE LSM was 14.5%. NAFLD prevalence increased with age, was higher among men than women and among Hispanics compared with non-Hispanic whites. Individuals who were obese, had metabolic syndrome (MetS) and type 2 diabetes were more likely to have NAFLD compared to those that who were not obese or without MetS/diabetes. Inadequate physical activity (OR = 1.57, 95% CI: 1.18–2.08) was also a factor associated with NAFLD. MetS, high waist circumstance, diabetes and hypertension were independently associated with significant NAFLD fibrosis.

**Conclusions:**

NAFLD and significant NAFLD fibrosis are highly prevalent in U.S. general population.

## Introduction

Nonalcoholic fatty liver disease (NAFLD) is the leading cause of chronic liver disease in the U.S. [1], and anticipated to become the leading indication for liver transplantation [2]. NAFLD is characterized by hepatic fat accumulation [3] and is the main hepatic complication of obesity and the metabolic syndrome [4]. NAFLD is a spectrum of disease ranging from steatosis to steatohepatitis with progressive fibrosis to cirrhosis. While most individuals with NAFLD are likely to have a good prognosis [5], up to 25% of NAFLD patients develop nonalcoholic steatohepatitis (NASH) [6] and 20% of NASH patients develop significant fibrosis (Metavir stage ≥2) [7], which is strongly associated with risk of adverse liver related complications, including hepatocellular carcinoma (HCC) [8].

Most population-based prevalence studies have relied on liver enzymes or ultrasonography for identifying and quantifying the burden of NAFLD; however, both methods potentially underestimate the true population prevalence of NAFLD [9, 10]. The sensitivity of using liver enzymes for NAFLD is low as liver enzymes may be normal in up to 78% of patients with NAFLD [11], while ultrasonography has a sensitivity of only 60%-94% and specificity of 66%-95% for detecting fatty liver [12]. Furthermore, neither method can quantify NAFLD fibrosis stage, which is essential for identifying high risk NAFLD cases. Vibration controlled transient elastography (VCTE) can estimate liver fibrosis by measuring liver stiffness; simultaneously it can quantify liver fat using the controlled attenuation parameter (CAP) with a sensitivity of 87% and specificity of 91% for detecting hepatic steatosis [13]. VCTE has been approved by the FDA as a test for the evaluation of liver fibrosis and is recommended in the current NAFLD clinical practice guidelines from the European Association for the Study of the Liver (EASL), European Association for the Study of Diabetes (EASD) and European Association for the Study of Obesity (EASO) [14].

There are no estimates for NAFLD prevalence or severity by CAP in the U.S. The National Health and Nutrition Examination Survey (NHANES), a survey among a nationally representative sample of the U.S. general population, used VCTE as part of its study procedures for the first time in 2017–2018. Using this more sensitive diagnostic technique will provide the best estimate yet for the population-based burden of NAFLD in the U.S., as well as factors associated with NAFLD. Analyses using these data so far have estimated a 47.8% age adjusted prevalence of hepatic steatosis (without excluding alcohol-related liver disease and defined as CAP≥263 dB/m) and 24.2% prevalence of NAFLD among adolescents [15]. However, NAFLD prevalence among the adult using this sensitive diagnostic technique is still unknown.

We therefore undertook this analysis using NHANES 2017–2018 survey data to: 1) estimate NAFLD prevalence; 2) estimate prevalence of significant NAFLD fibrosis; and 3) characterize the factors associated with NAFLD and significant NAFLD fibrosis in the U.S.

## Materials and methods

### Data source

We conducted a cross-sectional study using aggregated data from the 2017–2018 cycle of NHANES, a stratified, multistage probability sample representative of the civilian non-institutionalized U.S. population. NHANES methodology and data collection have been fully described previously [16] and are available on the NHANES website (http://www.cdc.gov/nchs/nhanes.htm). In brief, participants complete a survey capturing demographic, socioeconomic, dietary, and health-related information and a medical exam including anthropometric measurements and laboratory assessments. The National Center for Health Statistics institutional review board approved the overall NHANES and all participants provided written consent. The University of Texas, MD Anderson Cancer Center Institutional Review Board approved this analysis.

### Study population

**Fig 1:**
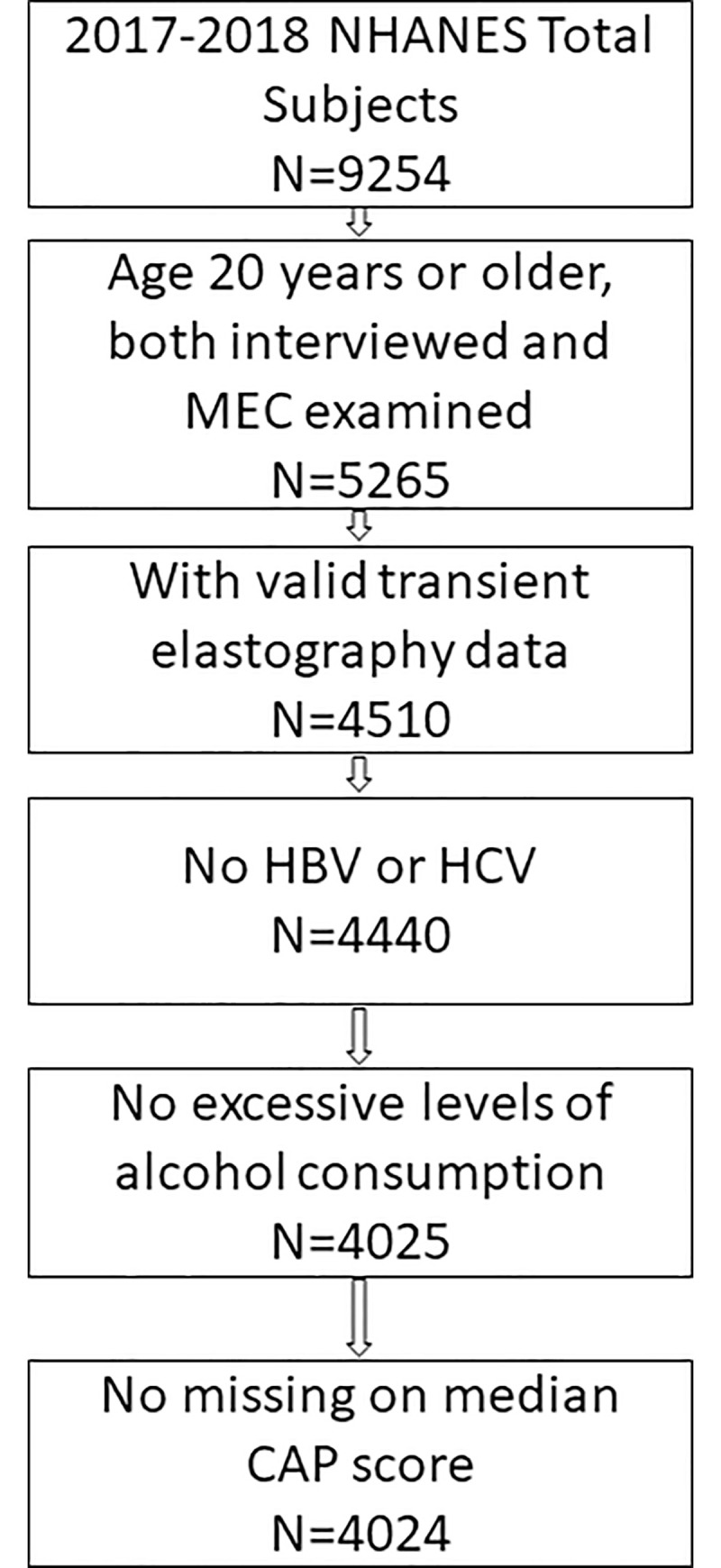
Flow chart for study population selection.

A total of 5,265 adults (age ≥20 years) participated in the 2017–2018 NHANES cycle and completed both the survey and medical examination. We excluded participants who did not undergo VCTE or with incomplete VCTE data (n = 755, due to partial exam, ineligibility or not done), or missing Median CAP scores (n = 1). We also excluded participants with evidence of alternative liver disease etiologies: hepatitis B surface antigen positivity (n = 27), hepatitis C antibody positivity (n = 43) and harmful alcohol drinking (≥30g/day for men or ≥ 20g/day for women; calculated using the dietary total nutrients data; n = 415). The final analysis sample included 4,024 participants (Fig 1). S1 Table compares our final study population with the source population (n = 5,265). We also compared the characteristics of those with successful VCTE measurement and those that failed VCTE measurement and found that obese participants were more likely to have failed VCTE measurement. There were no significant difference by age, gender, race and diabetes status (S2 Table).

### NAFLD and fibrosis definitions

NAFLD and NAFLD fibrosis were assessed using data obtained by VCTE with controlled attenuation. The VCTE measurements were obtained in the NHANES Mobile Examination Center (MEC), using the FibroScan® model 502 V2 Touch equipped with a medium (M) or extra-large (XL) wand (probe). For all examinations, the M probe was applied first; however, the operator switched to the XL probe if needed based on the recommendations of the device and the manufacturer’s instructions (M probe: Liver is ≤25 mm below skin; XL probe: liver is >25 mm below skin). The operator obtained a minimum of 10 measurements from each participant, and the device calculated the median CAP and LSM values along with the interquartile range. All studies were read over by a trained NHANES health technician to ensure quality. Exams were considered complete if participants fasted at least 3 hours prior to the exam, there were 10 or more complete LSM, and the liver stiffness IQR/median <30% [17]. The detailed procedure manual was described in the Liver Ultrasound Transient Elastography Procedures Manual [18]. In our final study population, 73% of participants used M probe, while 27% used XL probe. VCTE derives liver stiffness measurements (LSM) from the velocity of liver tissue microdisplacements induced by propagated shear waves. LSM measurements range from 1.5 kPa to 75kPa, with higher values indicating more severe fibrosis. Simultaneously, VCTE measures the CAP value, which reflects the ultrasonic attenuation in the liver. CAP values range from 100-400dB/m, with higher values indicating higher amounts of liver fat.

We defined NAFLD as a CAP score ≥248dB/m, based on published data from a large meta-analysis assessing CAP diagnostic cutoffs for NAFLD [19] and as used in previous population based studies [15]. In addition, we categorized patients into 3 steatosis severity levels: mild defined as CAP 248 to <268 dB/m (correlates with 10%-33% steatosis); moderate, CAP 268 to <280 dB/m (33%-66% steatosis); and severe, CAP ≥280 dB/m (≥66% steatosis) [19]. Individuals with CAP score <248 dB/m were considered non-NAFLD controls.

We defined NAFLD fibrosis according to published VCTE LSM cut-off values: F0-F1, <7.9 kPa; F2, 7.9 to <8.8 kPa; F3, 8.8 to <11.7 kPa; F4, ≥11.7 kPa [20]. NAFLD participants with a VCTE LSM value of 7.9 kPa or greater (≥F2) were considered to have significant NAFLD fibrosis [21]. We examined NAFLD fibrosis as a categorical variable (F01,2,3,4) and as a dichotomous variable (absence vs. presence of significant fibrosis).

We used the FibroScan–AST (FAST) score, a probability score that combines FibroScan results with an easily accessible blood biomarker to help identify patients with active fibrotic NASH (histologic nonalcoholic steatohepatitis (NASH), a NAFLD activity score≥ 4, and significant fibrosis (F≥2). Active Fibrotic NASH is identified by a cutoff of 0.35 for sensitivity of 0.90 or greater and a cutoff of 0.67 for specificity of 0.90 or greater [22]. In this study, we used both these two thresholds to detect active fibrotic NASH.

In order to make comparisons with prior NHANES analyses that relied on liver enzymes for NAFLD case definitions, we additionally assessed NAFLD based on liver enzyme cutoffs: aspartate aminotransferase (AST)> 37 or alanine aminotransferase (ALT)> 40 U/L in males or AST or ALT > 31 U/L in females [23].

### Measurement

#### Interview and biochemistry

The interview obtained information on age, sex, race/ethnicity, and household income. Physical activity was collected with the Global Physical Activity Questionnaire (GPAQ) developed by the World Health Organization [24]. Physical activity was classified as adequate versus inadequate physical activity. Adequate was defined as meeting the Physical Activity Guidelines for Americans: engaging in at least 150 minutes a week of moderate-intensity or 75 minutes a week of vigorous-intensity aerobic physical activity or an equivalent combination of moderate- and vigorous-intensity aerobic physical activity [25]. Inadequate was defined as anything less than meeting these guidelines. We estimated intake of energy nutrients and other food components using data collected as a part of the Dietary Recall Interview that assessed the food and beverage consumed by the participants during a 24-hour period before the interview. Laboratory methods for measurements of ALT and AST are reported in detail elsewhere [26].

#### Metabolic factors and comorbidities

Trained staff measured participants’ weight and height, as well as waist circumference. We calculated body mass index (BMI) as weight divided by height squared (kg/m^2^). Overweight status was defined as BMI 25 to 29.9 kg/m^2^ and obesity as BMI ≥ 30 kg/m^2^, while underweight was defined as BMI<18.5. High waist circumference was defined as waist circumference >102 cm in men and >88 cm in women. Hypertension was defined as systolic BP ≥ 130, diastolic BP ≥ 80 or ever told by a doctor they had high blood pressure or taking hypertension medications [27]. LDL cholesterol was calculated using the Friedewald formula [28]. Hyperlipidemia (HL) was defined as a serum cholesterol level of ≥200 mg/dL, LDL of ≥130 mg/dL, and HDL ≤ 40 mg/dL in men or ≤ 50 mg/dL in women. Diabetes was categorized as: normal [HgbA1C (<5.7%) and no self-report diabetes], pre-diabetes [HgbA1C (5.7–6.4%) and no self-report diabetes], and diabetes [HgbA1C (≥6.5%) or self-report diabetes]. The diagnosis of metabolic syndrome required the presence of three of the five measures which were created as binary affirmative variables according to the Adult Treatment Panel III criteria [29].

### Statistical analysis

Descriptive statistics were used to summarize data. We calculated NAFLD prevalence among the overall population; and significant NAFLD fibrosis prevalence among participants who had NAFLD by CAP. For between group comparisons, we used two sample t-test or Wilcoxon rank-sum test for continuous variables and Chi-Square test or Fisher’s exact test for categorical variables. Univariable linear regression models were used to examine difference across ordinal categories of steatosis grade and fibrosis stage. We used univariate and multivariate logistic regression models to assess predictors of NAFLD among the general population and predictors of significant fibrosis among participants with NAFLD. Variables selected for assessment were determined *a priori* based on clinical variables expected to be associated with NAFLD and fibrosis. In addition, we recognize that there is not currently a standard cut-off. Therefore, we have added a number of sensitivity analyses using different CAP cut-offs (290 dB/m [30] and 302 dB/m [31]) to define NAFLD.

Weighted analyses were carried out using survey weights, which was fundamental to NHANES. These weights were used to account for the complex survey design, survey non-response, post-stratification, and oversampling. By weighting, the sample becomes representative of the U.S. non-institutionalized population [32]. We used SAS 9.4 (SAS Institute INC, Cary, NC) for data analysis, and p <0.05 was used for statistical significance.

## Results

### Study population

The overall study population had a mean age of 48.4 years (SE, 0.6 years), 49% were male, and 61% were non-Hispanic white, 12% were non-Hispanic Black and 16% were Hispanic. Overall, 42.8% of participants were obese, 35.8% had pre-diabetes or diabetes, 26.6% had metabolic syndrome, and 35.9% of participants reported inadequate physical activity. Other study population characteristics are shown in Table 1 and S3 Table.

**Table 1:** Characteristics of factors according to NAFLD status by CAP.

Variables		Total		NAFLD Status*				
				Yes (CAP≥248 dB/m)		No (CAP<248 dB/m)		P-value
				(n = 2373)		(n = 1651)		
		n	Weighted % ± SE	n	Weighted % ± SE	n	Weighted % ± SE	
**Fibroscan CAP value (dB/m), Mean** ± **SE**								<0.001
		4024	263.6 ± 1.8	2373	308.3 ± 1.6	1651	205.1 ± 1.2	
**Age**								<0.001
	Mean ± SE	4024	48.4 ± 0.6	2373	51.4 ± 0.6	1651	44.5 ± 0.8	
	20–29	587	18.0 ± 1.3	230	11.8 ± 1.2	357	26.2 ± 2.2	
	30–39	617	17.9 ± 0.7	310	16.1± 1.1	307	20.3 ± 1.4	
	40–49	585	15.4 ± 0.9	355	15.2 ± 1.1	230	15.8 ± 1.4	
	50–59	691	19.2 ± 1.3	472	23.1 ± 2.0	219	14.0 ± 1.8	
	60–69	844	16.0 ± 1.5	570	18.1 ± 1.1	274	13.3 ± 2.0	
	70–79	450	9.6 ± 0.7	299	11.9 ± 1.1	151	6.4 ± 0.6	
	80–89	250	3.9 ± 0.4	137	3.8 ± 0.4	113	4.0 ± 0.5	
**Sex**								0.001
	Male	1941	48.5 ± 1.0	1226	53.5 ± 1.7	715	41.9 ± 1.7	
	Female	2083	51.5 ± 1.0	1147	46.5 ± 1.7	936	58.1 ± 1.7	
**Race**								<0.001
	Non-Hispanic White	1335	61.3±2.7	802	61.4±2.9	533	61.2±3.1	
	Non-Hispanic Black	940	11.6±1.7	465	9.5±1.7	475	14.4±1.8	
	Hispanics	938	16.4±2.1	638	18.3±2.5	300	13.7±1.8	
	Other	811	10.8±1.4	468	10.8±1.4	343	10.7±1.5	
**Household income**								0.65
	<$55,000	1948	41.7 ± 1.8	1146	42.4 ± 2.1	802	40.9 ± 2.8	
	≥$55,000	1704	58.2 ± 1.8	997	57.6 ± 2.1	707	59.1 ± 2.8	
**Smoking**								0.0944
	Nonsmoker	2404	60.1 ± 1.8	1388	57.8 ± 2.2	1016	63.0 ± 2.3	
	Former smoker	142	3.3 ± 0.4	88	3.8 ± 0.5	54	2.7 ± 0.5	
	Current smoker	1478	36.6 ± 1.7	897	38.3 ± 2.1	581	34.3 ± 2.3	
**Alcohol drinking**								0.0934
	Yes	638	20.6 ± 1.2	353	18.5 ± 1.8	285	23.3 ± 1.7	
	No	3036	79.4 ± 1.2	1810	81.4 ± 1.8	1226	76.7 ± 1.7	
**Physical activity**								
	Inadequate	1323	35.9 ± 1.4	847	41.8 ± 2.3	476	28.2 ± 1.3	<0.001
	Adequate	1750	64.1 ± 1.4	947	58.2 ± 2.3	803	71.8 ± 1.3	
**Carbohydrate intake (Mean ± SE)**		3674	240.6 ± 2.9	2163	245.8 ± 3.7	1511	234.0 ± 3.4	0.02
**Body mass index**								<0.001
	Mean ± SE	3991	29.7 ± 0.3	2351	32.7 ± 0.3	1640	25.9 ± 0.3	
	Underweight (<18.5)	58	1.5 ± 0.3	6	0.3 ± 0.2	52	3.1 ± 0.6	
	Normal (18.5 to 25)	990	24.8 ± 1.5	250	8.9 ± 0.8	740	45.7 ± 2.5	
	Overweight (25–29.9)	1304	30.8 ± 1.3	767	30.1 ±2.0	537	31.8 ± 1.6	
	Obesity (≥30)	1639	42.8 ± 2.1	1328	60.7 ± 2.3	311	19.4 ± 2.3	
**Diabetes**								<0.001
	Normal	2132	64.2 ± 1.2	992	52.1 ± 1.7	1140	79.8 ± 1.4	
	Pre-diabetes	993	21.7 ± 0.9	660	26.5 ± 1.1	333	15.5 ± 1.1	
	Diabetes	791	14.1 ± 0.6	647	21.4 ± 0.9	144	4.7 ± 0.6	
**Metabolic Syndrome**								<0.001
	Yes	1153	26.6 ± 1.3	977	40.2 ± 1.5	176	8.7 ± 0.8	
	No	2871	73.4 ± 1.3	1396	59.8 ± 1.5	1475	91.3 ± 0.8	
**NAFLD defined by liver enzymes**								0.002
	Yes	464	12.4 ± 0.8	348	15.9 ± 1.1	116	7.8 ± 1.3	
	No	3326	87.6 ± 0.8	1909	84.1 ± 1.1	1417	92.1 ± 1.3	
**AST (IU/L) (Mean± SE)**		3790	21.6 ± 0.2	2257	22.2 ± 0.4	1533	20.8 ± 0.5	0.051
**ALT (IU/L) (Mean± SE)**		3799	22.7 ± 0.4	2262	25.5 ± 0.6	1537	19.1 ± 0.4	<0.001
**VCTE LSM value (kPa, Mean± SE)**		4024	5.7 (0.1)	2373	6.4 (0.2)	1651	4.8 (0.1)	<0.001

### Prevalence of NAFLD

Approximately 57% of participants had NAFLD by CAP (weighted prevalence, 56.7%; 95% CI 53.5%-59.9%), corresponding to 102 million U.S. adults over 20 years of age, 95%CI: (96 million-108 million). The prevalence of S1, S2 and S3 steatosis by CAP were 9.9%, 7.7% and 39.1%, respectively (Table 2). When stratified by sex and race/ethnicity, males (62.6%) and Hispanics (63.7%) had higher NAFLD prevalence compared with females (48.8%) and other race/ethnicities (non-Hispanic white, 56.8%; non-Hispanic Black, 46.2%), respectively. The prevalence was highest in males aged 50–59 years (75.5%, 95% CI: 65.0%-86.0%) and females aged 70–79 years (68.7%, 95% CI: 60.2%-77.2%) (Tables 2 and 3). The sensitivity analysis using CAP cut-offs of 290 dB/m and 302 dB/m for NAFLD showed similar findings (S5 Table).

**Table 2:** Prevalence of steatosis stage, NAFLD defined by CAP, NAFLD defined by serum liver enzymes, fibrotic NASH defined by FAST (Fibroscan-AST) score and NAFLD fibrosis stage among NAFLD participants defined by VCTE LSM.

Steatosis Stage by CAP	N	%	95% CI
S0 (<10% steatosis, <248 dB/m)	1651	43.3	40.1–46.5
S1 (10%-33% steatosis, 248–268 dB/m, mild)	436	9.9	8.4–11.5
S2 (33%-66% steatosis, 268–280 dB/m, moderate)	314	7.7	6.0–9.3
S3 (≥66% steatosis, ≥280 dB/m, significant)	1623	39.1	36.6–41.7
**NAFLD defined by Steatosis (CAP≥248 dB/m)**			
No	1651	43.3	40.1–46.5
Yes	2373	56.7	53.5–59.9
**NAFLD defined by serum liver enzymes (AST > 37 or ALT > 40 U/L in males or AST or ALT > 31 U/L in females)**			
No	3326	87.6	86.0–89.2
Yes	464	12.4	10.8–14.0
**Fibrotic NASH ((NASH+NAS≥4+F≥2)**			
**Using cut-off point of 0.35**			
No	3520	93.6	92.5–94.6
Yes	270	6.4	5.4–7.5
**Using cut-off point of 0.67**			
No	3738	98.6	98.2–98.9
Yes	52	1.4	1.1–1.8
**NAFLD Fibrosis stage by VCTE LSM (Among NAFLD participants defined by CAP)**			
F0-F1 (<7.9 kPa)	2015	85.5	82.6–88.3
F2 (7.9 to <8.8 kPa)	88	3.9	2.2–5.6
F3 (8.8 to <11.7 kPa)	134	5.3	3.4–7.2
F4 (≥11.7 kPa)	136	5.3	4.2–6.5

**Table 3:** Weighted prevalence of NAFLD using the two definitions by age group, sex and race/ethnicity.

Sex by age		Non-Hispanic White		Non-Hispanic Black		Hispanics		Total	
		Prevalence	95% CI	Prevalence	95% CI	Prevalence	95% CI	Prevalence	95% CI
**NAFLD by CAP**									
**Male**		63.4	57.3, 69.5	45.6	39.0, 52.2	70.7	64.0, 77.4	62.6	58.0, 67.2
	20–29	41.2	25.8, 56.6	25.9	10.1, 41.6	56.9	39.3, 74.5	43.3	33.7, 52.8
	30–39	57.0	37.9, 76.2	37.8	25.1, 50.5	64.3	51.8, 76.9	56.9	47.4, 66.4
	40–49	58.1	38.1, 78.0	59.7	41.1, 78.3	84.9	71.4, 98.3	62.4	48.5,76.3
	50–59	77.0	63.7, 90.2	64.6	52.5, 76.7	80.9	70.5, 91.3	75.5	65.0, 86.0
	60–69	73.5	62.0, 85.0	54.2	43.7, 64.7	77.9	69.9, 85.9	71.9	63.4, 80.5
	70–79	74.3	63.9, 84.7	47.8	35.9, 59.8	86.0	72.8, 99.2	74.0	65.5, 82.5
	80–89	62.0	52.0, 72.1	30.1	13.3, 46.8	73.0	41.1, 100.0	61.2	52.1, 70.3
**Female**		50.4	44.3, 56.5	46.7	43.0, 50.3	57.0	51.6, 62.5	48.8	45.1, 52.6
	20–29	28.2	13.8, 42.7	19.0	8.9, 29.1	42.9	29.7, 56.1	30.5	22.1, 38.9
	30–39	47.1	35.1, 59.1	38.6	28.3, 48.9	52.8	41.6, 63.9	45.2	39.1, 51.3
	40–49	46.0	32.5, 59.4	48.2	31.3, 65.2	54.7	42.7, 66.8	49.6	41.5, 57.6
	50–59	57.9	41.0, 74.7	66.1	57.8, 75.1	68.9	58.8, 79.0	61.7	50.1, 73.3
	60–69	51.7	41.1, 62.3	63.6	54.1, 73.1	75.3	64.6, 86.1	56.8	48.5, 65.2
	70–79	70.4	60.4, 80.4	57.6	45.9, 69.3	65.2	51.7, 78.6	68.7	60.2, 77.2
	80–89	52.7	46.0, 59.3	35.2	9.9, 60.6	51.1	0.0–100.0	49.8	44.6, 55.0
**Total**		56.8	51.6, 61.9	46.2	42.5, 49.9	63.7	60.7, 66.7	56.7	53.5, 59.9
**NAFLD by elevated serum liver enzymes**									
**Male**		13.8	10.4, 17.2	13.3	9.5, 17.2	23.0	18.2, 27.7	15.3	12.8, 17.9
	20–29	29.2	17.8, 40.5	10.0	1.1, 18.8	28.0	14.8, 41.3	25.6	19.4, 31.7
	30–39	11.9	6.0, 17.8	20.8	7.8, 33.7	22.2	8.2, 26.3	17.0	10.5, 23.4
	40–49	15.9	2.4, 29.3	15.4	3.3, 27.5	34.5	17.5, 51.5	19.0	9.5, 28.5
	50–59	13.9	2.2, 25.6	15.6	5.9, 25.3	16.6	7.7, 25.5	13.9	6.6, 21.2
	60–69	7.7	0.9, 14.6	9.5	4.7, 14.3	8.2	2.8, 13.5	7.5	2.6, 12.4
	70–79	4.6	0.0, 10.1	3.6	0.0, 10.5	12.3	0.0, 26.8	4.9	0.8, 9.1
	80–89	3.4	0.0, 7.7	0.0	0.0–0.0	14.4	0.0, 38.6	4.4	0.6, 8.2
**Female**		8.8	6.2, 11.3	6.7	5.0, 8.5	12.6	9.8, 15.4	9.7	8.0, 11.4
	20–29	12.2	3.1, 21.3	1.9	0.0, 4.5	10.8	0.7, 20.9	9.8	4.3, 15.3
	30–39	10.2	2.8, 17.7	3.6	0.0, 7.9	14.2	7.5, 20.9	9.4	5.9, 13.0
	40–49	7.0	0.0, 15.7	7.0	1.0, 13.0	3.9	0.0, 8.0	7.4	2.6, 12.2
	50–59	8.6	2.2, 15.0	11.6	5.3, 17.8	17.8	9.9, 25.6	12.5	8.6, 16.4
	60–69	8.2	1.3, 15.1	9.4	3.9, 14.8	23.6	14.7, 32.5	10.4	5.5, 15.3
	70–79	9.1	1.4, 16.9	11.3	0.2, 22.3	7.3	0.0, 22.8	9.9	3.3, 16.5
	80–89	2.4	0.0, 6.0	4.9	0.0, 14.0	0	NA	2.5	0.0, 5.6
**Total**		11.2	8.8, 13.7	9.7	7.6, 11.7	17.6	15.1, 20.1	12.4	10.8, 14.0

The prevalence of NAFLD defined as elevated liver enzymes was 12.4%, corresponding to 21 million U.S. adults over 20 years of age, 95%CI: (18 million-24 million). When stratified by sex and race/ethnicity, males (15.3%) and Hispanics (17.6%) again had higher NAFLD prevalence compared with females (9.7%) and non-Hispanic whites (11.2%), respectively. In contrast to the NAFLD by CAP findings, prevalence was highest in males aged 20–29 years (25.6%, 95% CI: 19.4%-31.7%) and females aged 50–59 years (12.5, 95% CI: 8.6%-16.4%) (Tables 2 and 3).

### Factors associated with NAFLD

Table 4 shows the factors associated with NAFLD by CAP in univariate and multivariable analysis. In the multivariable analysis, age 50–59 years old was associated with 3-fold higher odds for NAFLD, compared with age 20–29 years old (OR = 3.13, 95% CI: 1.78–5.50). Compared with non-Hispanic whites, non-Hispanic Blacks had lower odds (OR = 0.73, 95% CI: 0.60–0.89) and Hispanics had higher odds (OR = 1.57, 95%CI: 1.23–2.01) for NAFLD. Metabolic syndrome (OR = 5.51, 95% CI: 4.37–6.94) and obesity (OR = 19.10, 95% CI: 11.16–32.69) were independently associated with increased odds for NAFLD. An increased odds was also seen in participants with prediabetes and diabetes. In addition, inadequate physical activity was associated a higher odds for NAFLD (OR = 1.57, 95% CI: 1.18–2.08). Factors associated with NAFLD by elevated liver enzymes are shown in S4 Table. Similar to the findings for NAFLD by CAP, in multivariable analysis, metabolic syndrome, obesity, hypertension, hyperlipidemia age, and sex were associated with NAFLD by elevated liver enzymes, while race/ethnicity and diabetes were not significantly associated with NAFLD by elevated liver enzymes. The findings were similar in the sensitivity analysis using CAP cut-offs of 290 dB/m and 302 dB/m for NAFLD (S6 and S7 Tables).

**Table 4:** Multivariable analysis for factors associated with NAFLD by CAP.

Variables		Crude OR	95%CI	Multivariable adjusted OR^a^	95%CI
**Age**					
	1 unit increase	1.02	1.018–1.030		
	20–29	1.76	1.20–2.56	Ref	
	30–39	2.13	1.47–3.10	1.25	0.81–1.93
	40–49	3.63	2.20–6.00	**1.82**	**1.18–2.80**
	50–59	2.99	2.12–4.23	**3.13**	**1.78–5.50**
	60–69	4.14	2.67–6.42	**2.94**	**1.88–4.59**
	70–79	2.07	1.48–2.88	**3.02**	**1.76–5.19**
	80–89	1.76	1.20–2.56	**1.54**	**1.10–2.14**
**Sex**					
	Male	Ref		Ref	
	Female	0.63	0.49–0.80	**0.61**	**0.44–0.83**
**Race**					
	Non-Hispanic White	Ref		Ref	
	Non-Hispanic Black	0.65	0.50–0.85	**0.73**	**0.60–0.89**
	Hispanics	1.34	1.04–1.71	**1.57**	**1.23–2.01**
	Other	1.00	0.76–1.32	1.18	0.97–1.43
High waist circumference^**^					
	1 unit increase	1.10	1.09–1.11	
	Yes	5.68	4.75–6.79	
	No	Ref		
Body mass index^*^					
	1 unit increase	1.23	1.20–1.26		
	Underweight (<18.5)	0.55	0.14–2.20	**1.44**	**0.42–4.88**
	Normal (18.5 to 25)	Ref		Ref	
	Overweight (25–29.9)	4.87	3.70–6.41	**5.00**	**2.96–8.43**
	Obesity (≥30)	16.13	11.04–23.59	**19.10**	**11.16–32.69**
Hyperlipidemia^*^					
	Yes	2.25	1.49–3.39	1.45	0.82–5.56
	No	Ref		Ref	
Diabetes^*^					
	Normal	Ref		Ref	
	Pre-diabetes	2.62	2.07–3.32	**1.93**	**1.32–2.84**
	Diabetes	6.95	4.91–9.84	**3.80**	**2.41–5.98**
**Metabolic Syndrome**					
	Yes	7.00	5.77–8.50	**5.51**	**4.37–6.94**
	No	Ref		Ref	
Hypertension^*^					
	Yes	2.75	2.24–3.37	**1.24**	**1.01–1.52**
	No	Ref		Ref	
**Smoking**					
	Nonsmoker	Ref			
	Former smoker	1.51	0.97–2.37		
	Current smoker	1.22	0.94–1.58		
**Alcohol drinking**					
	Yes	0.75	0.53–1.05		
	No	Ref			
**Physical activity**					
	**Inadequate**	1.83	1.44–2.33	**1.57**	**1.18–2.08**
	**Adequate**	Ref		Ref	
**Macronutrients**					
	**Average total energy intake (100 unit increase)**	1.16	1.03–1.30	0.48	0.13–1.72
	**Carbohydrate intake (10 unit increase)**	1.10	1.02–1.19	1.03	0.98–1.08
	**Total fat (10 unit increase)**	1.03	1.01–1.06	1.11	0.96–1.29

* Final model adjusted without metabolic syndrome.

** High waist circumference was not taken into final model due to high collinearity with obesity.

^a^ Final model including age, sex, race physical activity, total energy intake, carbohydrate intake, total fat with either metabolic syndrome or obesity, diabetes, hypertension, hyperlipidemia.

### Prevalence of significant NAFLD fibrosis

The prevalence of F2, F3 and F4 by VCTE LSM among patients with NAFLD by CAP were 3.9%, 5.3% and 5.3%, respectively (Table 2), and the prevalence of significant NAFLD fibrosis (≥F2) by VCTE LSM among patients with NAFLD by CAP was 14.5% (95% CI: 11.7%-17.4%), corresponding to 15 million U.S. adults over 20 years of age, 95%CI: (12 million-17 million). Males had a higher prevalence of significant NAFLD fibrosis than females (15.5% vs 13.4%) and Hispanic had the highest prevalence compared to other races/ethnicities (weighted prevalence 15.4%, 95% CI: 10.4% -20.5%). In males, the highest prevalence of significant fibrosis was among those aged 20–29 years (16.7%, 95% CI: 9.9%-17.2%), while in females, participants 30–39 years old (18.4%) and 70–79 years old (17.0%) had the first and second highest prevalence (Table 5).

**Table 5:** Weighted prevalence of significant NAFLD fibrosis (F2, F3 and F4) by age group, sex and race/ethnicity.

Sex by age		Non-Hispanic White		Non-Hispanic Black		Hispanics		Total	
		Prevalence	95% CI	Prevalence	95% CI	Prevalence	95% CI	Prevalence	95% CI
**Male**		15.2	10.1, 20.4	13.7	9.2, 18.2	16.9	11.1, 22.6	15.5	11.9, 19.1
	20–29	30.3	11.5, 49.2	9.4	0.0, 19.7	8.0	0.6, 15.4	16.7	9.9, 17.2
	30–39	12.5	0.2, 24.8	10.7	2.8, 18.6	10.9	0.0, 26.2	12.1	3.1, 21.1
	40–49	17.8	0.8, 34.8	10.5	1.6, 19.4	13.8	0.0, 27.8	16.4	5.2, 27.7
	50–59	11.8	6.0, 17.6	16.4	8.6, 24.3	28.9	13.3, 44.5	16.3	11.7, 20.9
	60–69	12.2	4.3, 20.1	23.6	16.8, 30.4	23.5	14.2, 32.8	14.2	8.6, 19.7
	70–79	13.6	3.6, 23.6	9.4	0.0, 24.0	27.2	7.3, 47.1	14.4	7.3, 21.6
	80–89	15.1	2.4, 27.8	0.0	NA	40.0	12.6, 67.3	15.7	4.4, 26.9
**Female**		13.8	8.5, 19.2	15.3	10.9, 19.7	13.8	6.7, 20.8	13.4	9.8, 16.9
	20–29	21.2	2.8, 39.5	0.0	NA	0.0	NA	10.7	0.2, 21.3
	30–39	20.7	7.1, 34.3	18.5	4.4, 32.6	17.1	7.1, 27.1	18.4	10.8, 26.0
	40–49	6.7	0.0, 13.7	13.5	4.1, 22.8	12.2	0.0, 25.4	7.7	3.2, 12.2
	50–59	15.4	0.2, 30.7	23.3	13.8, 32.8	18.2	7.2, 29.3	15.3	6.3, 24.3
	60–69	8.3	0.0, 17.3	12.1	3.2, 21.0	21.3	8.0, 34.5	11.1	5.0, 17.2
	70–79	15.6	6.6, 24.6	15.0	0.0, 35.2	14.8	3.3, 26.2	17.0	9.2, 24.9
	80–89	9.8	0.5, 19.1	0.0	NA	0	NA	8.4	0.4, 16.5
**Total**		14.6	10.8, 18.5	14.6	11.2, 18.0	15.4	10.4, 20.5	14.5	11.7, 17.4

### Factors associated with significant NAFLD fibrosis

Table 6 shows the factors associated with significant NAFLD fibrosis in univariate and multivariable analysis. Metabolic syndrome (adjusted OR = 2.39, 95% CI: 1.83–3.12), diabetes (adjusted OR = 3.97, 95% CI: 2.50–6.29), high waist circumstance (adjusted OR = 2.61, 95% CI: 1.17–5.82) and hypertension (adjusted OR = 1.50, 95% CI: 1.09–2.08) were each independently associated with significant fibrosis. No other demographic, behavioral or metabolic syndrome components were statistically significantly associated with significant NAFLD fibrosis.

**Table 6:** Multivariable analysis for factors associated with significant NAFLD fibrosis among NAFLD participants by CAP.

Variables		Crude OR	95%CI	Multivariable adjusted OR^a^	95%CI
**Age**					
	1 unit increase	0.998	0.992–1.004		
	20–29	Ref		Ref	
	30–39	0.91	0.48–1.73	0.81	0.45–1.47
	40–49	0.73	0.38–1.41	0.60	0.32–1.15
	50–59	0.97	0.65–1.45	0.80	0.52–1.23
	60–69	0.75	0.45–1.27	0.63	0.37–1.07
	70–79	0.97	0.54–1.75	0.82	0.47–1.45
	80–89	0.72	0.30–1.71	0.59	0.23–1.51
**Sex**					
	Male	Ref		Ref	
	Female	0.84	0.59–1.20	0.82	0.59–1.14
**Race**					
	Non-Hispanic White	Ref		Ref	
	Non-Hispanic Black	1.00	0.65–1.55	1.10	0.69–1.74
	Hispanics	1.07	0.71–1.61	1.06	0.67–1.67
	Other	0.82	0.50–1.33	0.87	0.54–1.41
High waist circumference^*^					
	1 unit increase	1.06	1.04–1.08		
	Yes	2.18	1.19–3.99	**2.61**	**1.17–5.82**
	No	Ref		Ref	
**Body mass index**					
	1 unit increase	1.12	1.09–1.16		
	Underweight (<18.5)**	NA	-		
	Normal (18.5 to 25)	Ref			
	Overweight (25–29.9)	0.41	0.13–1.27		
	Obesity (≥30)	1.92	0.59–6.31		
Hyperlipidemia^*^					
	Yes	1.43	0.87–2.35	1.25	0.72–2.17
	No	Ref		Ref	
Diabetes^*^					
	Normal	Ref		Ref	
	Pre-diabetes	0.87	0.51–1.46	0.96	0.55–1.68
	Diabetes	3.27	2.36–4.54	**3.97**	**2.50–6.29**
**Metabolic Syndrome**					
	Yes	2.29	1.72–3.03	**2.39**	**1.83–3.12**
	No	Ref		Ref	
Hypertension^*^					
	Yes	1.62	1.19–2.20	**1.50**	**1.09–2.08**
	No	Ref		Ref	
**Smoking**					
	Nonsmoker	Ref			
	Former smoker	1.19	0.53–2.67		
	Current smoker	1.05	0.82–1.34		
**Alcohol drinking**					
	Yes	1.03	0.64–1.67		
	No	Ref			
**Physical activity**					
	**Inadequate**	1.21	0.74–1.99		
	**Adequate**	Ref			
**Macronutrients**					
	**Average total energy intake (1000 unit increase)**	1.15	0.86–1.54		
	**Carbohydrate intake (10 unit increase)**	0.99	0.80–1.21		
	**Total fat (10 unit increase)**	1.04	0.98–1.10		

* Final model adjusted without metabolic syndrome.

** The cell frequency is too small to obtain a OR.

^a^ Final model including age, sex, race with either metabolic syndrome or high waist circumference, diabetes, hypertension, hyperlipidemia.

### Prevalence of fibrotic NASH using FAST (Fibroscan-AST) score

Using a cutoff point of 0.35, the prevalence of fibrotic NASH is 6.4%, 95%CI: 5.4%-7.5%; while using a cutoff point of 0.67, the prevalence of fibrotic NASH is 1.4%, 95%CI: 1.1%-1.8% (Table 2).

## Discussion

This is the first study to report NAFLD prevalence and significant NAFLD fibrosis among U.S. adults using VCTE measurements. In this nationally representative, population-based cross-sectional study, approximately 57% of U.S. adults over 20 years of age during the 2017–2018 time period had NAFLD, suggesting 102 million U.S. adults over 20 years of age with NAFLD (CAP≥248 dB/m). Assuming a more stringent NAFLD thresholds (CAP≥290 dB/m or 302 dB/m), NAFLD is present in at least 51 million U.S. adults over 20 years of age. Among those with NAFLD, 15% had significant fibrosis, suggesting 15 million U.S. adults over 20 years of age with NAFLD also have significant fibrosis. Men, middle-aged adults and Hispanics had the highest prevalence rates of NAFLD, while non-Hispanic blacks had the lowest. Metabolic dysfunction (metabolic syndrome, obesity, diabetes) and inadequate physical activity were strongly and independently associated with NAFLD, while metabolic syndrome and diabetes were independently associated with significant fibrosis among those with NAFLD. Moreover, our findings are robust in regarding of the choice of CAP cut-off.

The 57% NAFLD prevalence that we detected by CAP is higher than previously reported among the U.S. population. NHANES based surveys have shown a gradual increase in NAFLD population prevalence over time: from 19.0% in NHANES 1988–1994 (diagnosed by ultrasonography) [33] to 32.2% in NHANES 1999–2016 (diagnosed by United States Fatty Liver Index (USFLI)>30) [34] to 53.6% in NHANES 2005–2016 (diagnosed by hepatic steatosis index (HSI)>36) [35]. The higher prevalence we observed may be explained to some degree by the fact that CAP is the most sensitive diagnostic measure used by so far NHANES cycle and we used the lowest CAP threshold for identification of hepatic steatosis [19]. However, the higher prevalence may also reflect a real increase in disease prevalence over time. In keeping with this possibility, we observed 12.4% NAFLD prevalence using the same liver enzyme cutoffs utilized by previous NHANES reports and represents the highest reported prevalence to date (vs. 5.5% in NHANES 1988–1994 to 9.8% in NHANES 1999–2004 and 11.0% in NHANES 2005–2008 [36].

The prevalence of significant NAFLD fibrosis among NAFLD participants using VCTE was 14.5%. This is higher than reported among previous NHANES surveys. For example, advanced fibrosis prevalence among NAFLD participants was estimated at 10.3% in NHANES 1988–1994 using the NAFLD fibrosis score [37]. Subsequently, the prevalence of advanced fibrosis among NAFLD participants was reported as increasing from 3.3% (2005–2008) and 6.4% (2009–2012), to 6.8% (2013) using the FIB4 and APRI scores [35]. The reason why our prevalence of significant fibrosis was higher than previous studies may be due to definitions: previous studies defined significant/advanced fibrosis using NFS, APRI or FIB4 [38]. In contrast, we defined significant fibrosis as ≥F2 using VCTE LSM because data over the years has shown that METAVIR stage 2 and higher is strongly associated with adverse liver disease outcomes among patients with NAFLD [39]. Our findings suggest that approximately 15 million American adults have significant NAFLD fibrosis and are at high risk for disease progression and complications, such as liver failure and HCC [39].

Consistent with prior analyses we found that males, older age, and Hispanic ethnicity were independently associated with NAFLD. The age trends for NAFLD are not strong in our findings compared to previous findings [33]. However, the age trends within ethnicities were notably different. The highest NAFLD prevalence rates were seen among non-Hispanic whites between 50 and 79 years of age. In contrast, NAFLD prevalence rates were higher at a younger age among Hispanics than non-Hispanic whites, which is in line with prior observations [40]. Given the rapidly increasing rate of NAFLD and obesity in younger people, there will be a substantial burden on the U.S. health system as they age. There was also a higher prevalence of NAFLD among Hispanics compared with other races/ethnicities in our study. This finding may be due to the high prevalence of *PNPLA3* (a gene associated with increased susceptibility to hepatic steatosis, NASH, and fibrosis) G allele among Hispanics and/or the high prevalence of metabolic factors [41]. Meanwhile, our prevalence of NAFLD was higher in males than females which is in keeping with the findings of a recent meta-analysis [42].

We confirmed that metabolic syndrome and diabetes are strong independent factors associated with both NAFLD and significant NAFLD fibrosis, in keeping with findings from prior analyses conducted among both NHANES and non-NHANES study populations [36, 43, 44]. Interestingly, we found that inadequate physical activity was associated with higher NAFLD prevalence independent of metabolic factors and BMI. This finding aligns with prior studies showing that patients with NAFLD exercise less frequently than those without NAFLD [45] and that most patients with NAFLD fail to meet the recommended physical activity guidelines [46]. While our study using CAP-defined NAFLD found associations with race/ethnicity and physical activity, these factors were not associated with NAFLD fibrosis when using a less accurate NAFLD definition by ALT/AST.

It is well established that NASH with significant fibrosis is the most concerning phenotype of NAFLD, accounting for a relatively small proportion of cases. Prior biopsy based studies have shown 12% prevalence of NASH fibrosis and constitutes a relatively small proportion [45]. In our study, using a cut-off of 0.35, the prevalence of fibrotic NASH was 6.4%; while using a cutoff point of 0.67, the prevalence of fibrotic NASH was 1.4%. The difference is probably because our study was a population based study, whereas most studies that report prevalence of significant NASH used biopsy-proven NAFLD study populations, which might introduce potential selection bias and overestimate the true prevalence of NASH.

To our knowledge, this is the first study reporting NAFLD and significant NAFLD fibrosis estimates among adults from the general U.S. population using VCTE and CAP. Current clinical evidence suggests that CAP and VCTE LSM had a high sensitivity and specificity to diagnosis liver disease from significant fibrosis to liver cirrhosis [47]. In this study, we used a CAP threshold of 248 dB/m to define NAFLD (S1 and higher). There is no universally agreed upon CAP cut point to identify NAFLD in population based studies. The cut points used in this study come from the largest meta-analysis (including 21 studies, and 2735 patients with NAFLD defined by biopsy) addressing the correlation between CAP cut point and hepatic steatosis; and they have been used in several studies [21, 48–50]. We used the lowest threshold in order to provide greater clarity into the prevalence of all 3 hepatic steatosis severity level in the population, which has never previously been described in the U.S. population. While using two more stringent cut-offs (CAP≥290 dB/m and ≥302 dB/m) gave similar findings. Meanwhile, we excluded other liver disease caused by HCV, HBV and excessive alcohol drinking. Finally, we focused on and included traditional factors associated with NAFLD and fibrosis that are supported by a large body of prior work. Our study has several limitations. First, while VCTE is reliable for distinguishing between presence versus absence of significant fibrosis, we acknowledge that it is less reliable for differentiating between F0 and F1 and for identifying discrete stages of fibrosis. Second, the cross-sectional study design limits causal inference. Third, obese participants were more likely to have failure of VCTE measurement, which might lead to potential selection bias in our final results. Fourth, notably, 23% of participants were measured using XL probe, as previously reported, the median liver stiffness might be significantly lower than that measured with the M probe [51]. Last, there may be some selection bias for the NHANES 2017–2018, as individuals with more significant forms of disease may not participate in NHANES; however, this bias would result in underestimating the true association between factors and NAFLD.

## Conclusions

NAFLD, as a body fat problem has affected approximately 57% of the U.S. population, and 15% of those with NAFLD have significant fibrosis. Overall, 6% of the U.S population have significant NASH detected by FAST (Fibroscan-AST) score with a cutoff point of 0.35. These are the people that are potentially the targets of pharmaceutical trials and agents for disease modification. Hispanics, middle to older adults, males, people with metabolic syndrome, diabetes, obesity, and low levels of physical activity are most affected. Therefore, multidimensional and precision public health programs should use race/ethnicity, sex, age, metabolic profile and behavioral patterns to target efforts to address NAFLD prevention and treatment.

## Supporting information

S1 Table Characteristics comparison between included and excluded participants. (DOCX)

S2 Table Characteristics comparison between participants with successful VCTE measurement and failed VCTE measurement. (DOCX)

S3 Table Characteristics of factors according to NAFLD status by CAP. (DOCX)

S4 Table Multivariable analysis for risk factors for NAFLD by elevated liver enzymes. (DOCX)

S5 Table Weighted prevalence of NAFLD using two cut off points by age group, sex and race/ethnicity. (DOCX)

S6 Table Multivariable analysis for factors associated with NAFLD by CAP cut off point of 290 dB/m. (DOCX)

S7 Table Multivariable analysis for factors associated with NAFLD by CAP cut off point of 302 dB/m. (DOCX)

S1 File Data file for this study. (XLSX)

## Abbreviations

HCC: hepatocellular carcinoma
AST: aspartate transaminase
ALT: alanine transaminase
NAFLD: Non-alcoholic fatty liver disease
CAP: controlled attenuation parameter
MetS: metabolic syndrome
NASH: nonalcoholic steatohepatitis
VCTE: Vibration controlled transient elastography
NHANES: National Health and Nutrition Examination Survey
LSM: liver stiffness measurements
GPAQ: Global Physical Activity Questionnaire
