# Supplementary material for: Prevalence and factors associated with NAFLD detected by vibration controlled transient elastography among US adults: Results from NHANES 2017–2018
Source: PLoS One. 2021 Jun 3;16(6):e0252164. doi: 10.1371/journal.pone.0252164 (PMC8174685; doi:10.1371/journal.pone.0252164)
Supplement: S1 Table — (DOCX) [file pone.0252164.s001.docx]

| **S1 Table**. Characteristics comparison between included and excluded participants. | | | | | | | | | | | |
| --- | --- | --- | --- | --- | --- | --- | --- | --- | --- | --- | --- |
| **Variables** | | **NHANES 2017-2018** | | | | | | | | | |
|  |  | **Included for CAP defined NAFLD** | | **Excluded for CAP defined NAFLD** | | **P-value** | **Included for serum liver enzymes  defined NAFLD** | | **Excluded for serum liver enzymes NAFLD** | | **P-value** |
|  |  | **(n=4024)** | | **(n=1241)** | |  | **(n=3790)** | | **(n=1475)** | |  |
|  |  | **n** | **Weighted % ± SE** | **n** | **Weighted % ± SE** |  | **n** | **Weighted % ± SE** | **n** | **Weighted % ± SE** |  |
| **Age** | |  |  |  |  |  |  |  |  |  |  |
|  | Mean ± SE | 4024 | 48.4 ± 0.6 | 1241 | 48.0 ± 0.9 | 0.73 | 3790 | 48.5 ± 0.7 | 1475 | 47.8 ± 0.8 | 0.51 |
| **Sex** | |  |  |  |  | 0.66 |  |  |  |  | 0.87 |
|  | Male | 1941 | 48.5 ± 1.0 | 600 | 47.1 ± 2.6 |  | 1810 | 48.2 ± 1.1 | 731 | 47.8 ± 2.3 |  |
|  | Female | 2083 | 51.5 ± 1.0 | 641 | 52.9 ± 2.6 |  | 1980 | 51.7 ± 1.1 | 744 | 52.2 ± 2.3 |  |
| **Race** | |  |  |  |  | 0.15 |  |  |  |  |  |
|  | Non-Hispanic White | 1335 | 61.3 ± 2.7 | 472 | 65.2 ± 2.8 |  | 1272 | 61.6 ± 2.6 | 535 | 63.8 ± 3.1 | 0.11 |
|  | Non-Hispanic Black | 940 | 11.6 ± 1.7 | 300 | 11.0 ± 1.8 |  | 852 | 11.0 ± 1.7 | 388 | 12.6 ±2.0 |  |
|  | Hispanics | 938 | 16.3 ± 2.1 | 256 | 14.1 ± 1.8 |  | 909 | 16.6 ± 2.1 | 285 | 13.7 ±1.8 |  |
|  | Other | 811 | 10.8 ± 1.4 | 213 | 9.8 ± 1.3 |  | 757 | 10.8 ±1.4 | 267 | 9.9 ±1.3 |  |
| **BMI** | |  |  |  |  |  |  |  |  |  |  |
|  | Mean ± SE | 3991 | 29.7 ± 0.3 | 1184 | 30.2 ± 0.4 | 0.26 | 3761 | 29.8 ± 0.4 | 1414 | 30.0 ± 0.4 | 0.52 |
