# Supplementary material for: Prevalence and factors associated with NAFLD detected by vibration controlled transient elastography among US adults: Results from NHANES 2017–2018
Source: PLoS One. 2021 Jun 3;16(6):e0252164. doi: 10.1371/journal.pone.0252164 (PMC8174685; doi:10.1371/journal.pone.0252164)
Supplement: S2 Table — (DOCX) [file pone.0252164.s002.docx]

| **S2 Table. Characteristics comparison between participants with successful VCTE measurement and failed VCTE measurement** | | | | | | |
| --- | --- | --- | --- | --- | --- | --- |
|  | | **Successful VCTE measurement** | | **Failed VCTE measurement** | | **P-value** |
|  |  | **(n=4024)** | | **(n=328)** | |  |
|  |  | **n** | **Weighted % ± SE** | **n** | **Weighted % ± SE** |  |
| **Age** | |  |  |  |  |  |
|  | Mean ± SE | 4024 | 48.4 ± 0.6 | 328 | 48.9 ± 1.4 | 0.71 |
| **Sex** | |  |  |  |  | 0.69 |
|  | Male | 1941 | 48.5 ± 1.0 | 156 | 53.2 ± 3.9 |  |
|  | Female | 2083 | 51.5 ± 1.0 | 172 | 48.5 ± 3.9 |  |
| **Race** | |  |  |  |  | 0.94 |
|  | Non-Hispanic White | 1335 | 61.3 ± 2.7 | 120 | 62.1 ± 5.0 |  |
|  | Non-Hispanic Black | 940 | 11.6 ± 1.7 | 81 | 12.4 ± 2.3 |  |
|  | Hispanics | 938 | 16.3 ± 2.1 | 75 | 15.6 ± 2.9 |  |
|  | Other | 811 | 10.8 ± 1.4 | 52 | 10.0 ± 2.9 |  |
| **BMI** | |  |  |  |  |  |
|  | Mean ± SE | 3991 | 29.7 ± 0.3 | 322 | 32.9 ± 0.9 | **0.0023** |
|  | Non-obesity | 2352 | 57.2 ± 2.1 | 144 | 47.4 ± 4.5 | **0.04** |
|  | Obesity | 1639 | 42.8 ± 2.1 | 178 | 52.6 ± 4.5 |  |
| **Diabetes** | |  |  |  |  | 0.15 |
|  | Normal | 2132 | 64.2 ± 1.2 | 155 | 56.3 ± 4.5 |  |
|  | Pre-diabetes | 993 | 21.7 ± 0.9 | 85 | 26.2 ± 4.0 |  |
|  | Diabetes | 791 | 14.1 ± 0.6 | 83 | 17.4 ± 2.9 |  |
