# Supplementary material for: Prevalence and factors associated with NAFLD detected by vibration controlled transient elastography among US adults: Results from NHANES 2017–2018
Source: PLoS One. 2021 Jun 3;16(6):e0252164. doi: 10.1371/journal.pone.0252164 (PMC8174685; doi:10.1371/journal.pone.0252164)
Supplement: S3 Table — (DOCX) [file pone.0252164.s003.docx]

| **S3 Table.** Characteristics of factors according to NAFLD status by CAP. | | | | | | | | |
| --- | --- | --- | --- | --- | --- | --- | --- | --- |
| **Variables** | | **Total** | | **NAFLD Status^*^** | | | | |
|  |  |  |  | Yes (CAP≥248 dB/m) | | No (CAP<248 dB/m) | | **P-value** |
|  |  |  |  | (n=2373) | | (n=1651) | |  |
|  |  | n | Weighted % ± SE | n | Weighted % ± SE | n | Weighted % ± SE |  |
| **High waist circumference** | | | | | | | | <0.0001 |
|  | **Mean ± SE** | 3869 | 100.6 ± 0.8 | 2277 | 108.7 ± 0.7 | 1592 | 90.0 ± 0.7 |  |
|  | Yes | 2285 | 58.1 ± 1.5 | 1711 | 75.5 ± 1.2 | 574 | 35.2 ± 2.1 |  |
|  | No | 1739 | 41.9 ± 1.5 | 662 | 24.5 ± 1.2 | 1077 | 64.8 ± 2.1 |  |
| **Total cholesterol >=240 mg/dL** | | | |  |  |  |  | 0.2832 |
|  | Yes | 398 | 10.7 ± 0.9 | 255 | 11.5 ± 1.1 | 143 | 9.6 ± 1.4 |  |
|  | No | 3422 | 89.3 ± 0.9 | 2018 | 88.5 ± 1.1 | 1404 | 90.4 ± 1.4 |  |
| **Low HDL-C** | |  | | | | | | <0.0001 |
|  | Yes | 1279 | 31.7 ± 1.6 | 960 | 41.1 ± 1.9 | 319 | 19.2 ± 1.4 |  |
|  | No | 2541 | 68.3 ± 1.6 | 1313 | 58.9 ± 1.9 | 1228 | 80.8 ± 1.4 |  |
| **Hyperlipidemia** | |  |  |  |  |  |  | 0.0005 |
|  | Yes | 241 | 5.9 ± 0.5 | 186 | 7.6 ± 0.7 | 55 | 3.5 ± 0.6 |  |
|  | No | 3579 | 94.1 ± 0.5 | 2087 | 92.4 ± 0.7 | 1492 | 96.5 ± 0.6 |  |
| **Hypertension** | |  |  |  |  |  |  | <0.0001 |
|  | Yes | 2183 | 49.5 ± 1.7 | 1480 | 60.2 ± 1.5 | 703 | 35.5 ± 2.4 |  |
|  | No | 1669 | 50.5 ± 1.7 | 781 | 39.8 ± 1.5 | 888 | 64.5 ± 2.4 |  |
| **Metabolic Syndrome** | |  | | | | | | <0.0001 |
|  | Yes | 1153 | 26.6 ± 1.3 | 977 | 40.2 ± 1.5 | 176 | 8.7 ± 0.8 |  |
|  | No | 2871 | 73.4 ± 1.3 | 1396 | 59.8 ± 1.5 | 1475 | 91.3 ± 0.8 |  |
| **Physical activity** | |  |  |  |  |  |  |  |
|  | **Total METs per week** **(Mean ± SE)** | 3073 | 4933. 7 ± 282.4 | 1794 | 4359.1 ± 303.5 | 1279 | 5670.5 ± 322.7 | 0.0003 |
| **Sedentary lifestyle** | |  |  |  |  |  |  | 0.6826 |
|  | Hours/day  (**Mean** ± **SE**) | 4016 | 6.7 ± 0.3 | 2367 | 6.5 ± 0.4 | 1649 | 6.8 ± 0.6 |  |
|  | Low tertiles | 1308 | 27.8 ± 1.8 | 742 | 27.2 ± 1.8 | 566 | 28.6 ± 2.1 |  |
|  | Medium tertiles | 1468 | 38.2 ± 1.2 | 879 | 38.1 ± 1.7 | 589 | 38.2 ± 1.7 |  |
|  | High tertiles | 1240 | 34.0 ± 1.6 | 746 | 34.7 ± 1.9 | 494 | 33.1 ± 1.8 |  |
| **Macronutrients** | |  |  |  |  |  |  |  |
|  | **Average total energy intake (Mean ± SE)** | 3674 | 2043.5 ± 18.8 | 2163 | 2090.1 ± 26.7 | 1511 | 1983.5 ± 24.7 | 0.0098 |
|  | **Average total protein intake (Mean ± SE)** | 3674 | 80.4 ± 1.1 | 2163 | 81.7 ± 1.4 | 1511 | 78.7 ± 1.7 | 0.1601 |
|  | **Fiber intake (Mean± SE)** | 3674 | 16.8 ± 0.4 | 2163 | 16.5 ± 0.4 | 1511 | 17.0 ± 0.5 | 0.2887 |
|  | **Total fat**  **(Mean ± SE)** | 3674 | 85.3 ± 0.8 | 2163 | 87.7 ± 1.2 | 1511 | 82.3 ± 1.2 | 0.0122 |
|  | **Total sugar**  **(Mean ± SE)** | 3674 | 103.7 ± 1.8 | 2163 | 105.2 ± 2.2 | 1511 | 101.8 ± 2.1 | 0.176 |
