# Supplementary material for: Prevalence and factors associated with NAFLD detected by vibration controlled transient elastography among US adults: Results from NHANES 2017–2018
Source: PLoS One. 2021 Jun 3;16(6):e0252164. doi: 10.1371/journal.pone.0252164 (PMC8174685; doi:10.1371/journal.pone.0252164)
Supplement: S4 Table — (DOCX) [file pone.0252164.s004.docx]

| **S4 Table.** Multivariable analysis for risk factors for NAFLD by elevated liver enzymes | | | | | |
| --- | --- | --- | --- | --- | --- |
| **Variables** | | **Crude OR** | **95%CI** | **Multivariable adjusted OR^a^** | **95%CI** |
| **Age** | |  |  |  |  |
|  | 1 unit increase | 0.98 | 0.97-0.99 |  |  |
|  | 20-29 | Ref |  | Ref |  |
|  | 30-39 | 0.69 | 0.49-4.97 | **0.59** | **0.42-0.82** |
|  | 40-49 | 0.68 | 0.39-1.21 | 0.57 | 0.31-1.06 |
|  | 50-59 | 0.69 | 0.45-1.05 | **0.55** | **0.34-0.89** |
|  | 60-69 | 0.45 | 0.26-0.77 | **0.38** | **0.22-0.66** |
|  | 70-79 | 0.38 | 0.16-0.89 | **0.32** | **0.14-0.71** |
|  | 80-89 | 0.16 | 0.07-0.36 | **0.14** | **0.06-0.33** |
| **Sex** | |  |  |  |  |
|  | Male | Ref |  | Ref |  |
|  | Female | 0.59 | 0.46-0.77 | **0.60** | **0.46-0.79** |
| **Race** | |  |  |  |  |
|  | Non-Hispanic White | Ref |  | Ref |  |
|  | Non-Hispanic Black | 0.85 | 0.61-1.19 | 0.84 | 0.61-1.14 |
|  | Hispanics | 1.69 | 1.24-2.32 | 1.42 | 1.04-1.93 |
|  | Other | 1.28 | 0.84-1.96 | 1.25 | 0.81-1.92 |
| **High waist circumference^**^** | |  |  |  |  |
|  | 1 unit increase | 1.02 | 1.01-1.03 |  |  |
|  | Yes | 1.56 | 1.19-2.03 |  |  |
|  | No | Ref |  |  |  |
| **Body mass index^*^** | |  |  |  |  |
|  | 1 unit increase | 1.05 | 1.03-1.08 |  |  |
|  | Underweight (<18.5) | 1.69 | 0.38-7.64 | 1.80 | 0.49-6.59 |
|  | Normal (18.5 to 25) | Ref |  | Ref |  |
|  | Overweight (25–29.9) | 1.19 | 0.84-1.69 | 1.05 | 0.75-1.48 |
|  | Obesity (≥30) | 2.51 | 1.58-4.00 | **1.99** | **1.34-2.97** |
| **Hyperlipidemia^*^** | |  |  |  |  |
|  | Yes | 1.98 | 1.11-3.53 | **1.94** | **1.12-3.36** |
|  | No | Ref |  | Ref |  |
| **Diabetes^*^** | |  |  |  |  |
|  | Normal | Ref |  | Ref |  |
|  | Pre-diabetes | 1.17 | 0.76-1.82 | 1.39 | 0.85-2.26 |
|  | Diabetes | 1.30 | 0.88-1.92 | 1.36 | 0.87-2.12 |
| **Metabolic Syndrome** | |  |  |  |  |
|  | Yes | 2.11 | 1.55-2.89 | **2.53** | **1.76-3.65** |
|  | No | Ref |  | Ref |  |
| **Hypertension^*^** | |  |  |  |  |
|  | Yes | 1.58 | 1.02-2.44 | **2.05** | **1.25-3.34** |
|  | No | Ref |  | Ref |  |
| **Smoking** | |  |  |  |  |
|  | Nonsmoker | Ref |  | Ref |  |
|  | Former smoker | 1.69 | 1.03-2.79 | 1.33 | 0.83-2.11 |
|  | Current smoker | 0.81 | 0.54-1.21 | 0.79 | 0.54-1.14 |
| **Alcohol drinking** | |  |  |  |  |
|  | Yes | 0.83 | 0.44-1.55 |  |  |
|  | No | Ref |  |  |  |
| **Physical activity** | |  |  |  |  |
|  | Inadequate | 0.81 | 0.60-1.09 |  |  |
|  | Adequate | Ref |  |  |  |
| **Macronutrients** | |  |  |  |  |
|  | **Average total energy intake  (1000 unit increase)** | 1.08 | 0.91-1.28 |  |  |
|  | **Carbohydrate intake  (10 unit increase)** | 1.00 | 0.86-1.17 |  |  |
|  | **Total fat (10 unit increase)** | 1.02 | 0.98-1.05 |  |  |
| ^*^ Final model adjusted without metabolic syndrome ^**^ High waist circumference was not taken into final model due to high collinearity with obesity.  ^a^ Final model including age, sex, race, smoking status, with either metabolic syndrome or obesity, diabetes, hypertension, hyperlipidemia. | | | | | |
|  |  |  |  |  |  |
