# Supplementary material for: Prevalence and factors associated with NAFLD detected by vibration controlled transient elastography among US adults: Results from NHANES 2017–2018
Source: PLoS One. 2021 Jun 3;16(6):e0252164. doi: 10.1371/journal.pone.0252164 (PMC8174685; doi:10.1371/journal.pone.0252164)
Supplement: S5 Table — (DOCX) [file pone.0252164.s005.docx]

| **S5 Table**. Weighted prevalence of NAFLD using two cut off points by age group, sex and race/ethnicity | | | | | | | | | |
| --- | --- | --- | --- | --- | --- | --- | --- | --- | --- |
| **Sex by age** | | **Non-Hispanic White** | | **Non-Hispanic Black** | | **Hispanics** | | **Total** | |
|  |  | **Prevalence** | **95% CI** | **Prevalence** | **95% CI** | **Prevalence** | **95% CI** | **Prevalence** | **95% CI** |
| **NAFLD by CAP cut off point of 290 dB/m** | | | | | | | | | |
| **Male** | | 40.4 | 34.0, 46.7 | 23.8 | 18.9, 28.7 | 47.0 | 41.4, 52.6 | 39.5 | 34.6, 44.5 |
|  | 20-29 | 25.6 | 10.2, 41.0 | 11.7 | 6.6, 16.8 | 40.0 | 21.5, 58.6 | 26.6 | 17.0, 36.1 |
|  | 30-39 | 27.5 | 14.4, 40.6 | 18.8 | 9.6, 28.0 | 43.4 | 28.6, 58.3 | 30.8 | 23.1, 38.6 |
|  | 40-49 | 42.9 | 24.2, 61.6 | 30.0 | 15.5, 44.5 | 54.3 | 37.2, 71.4 | 43.2 | 29.8, 56.5 |
|  | 50-59 | 47.2 | 32.3, 62.1 | 38.9 | 20.2, 57.6 | 52.9 | 41.7, 64.0 | 47.7 | 35.9, 59.5 |
|  | 60-69 | 48.6 | 34.4, 62.9 | 27.3 | 20.3, 20.3 | 48.3 | 38.2, 58.4 | 46.4 | 35.4, 57.4 |
|  | 70-79 | 52.0 | 40.3, 63.8 | 28.0 | 9.0, 47.1 | 61.1 | 42.9, 79.3 | 49.4 | 39.3, 59.5 |
|  | 80-89 | 41.3 | 30.2, 52.5 | 13.6 | 0.0, 27.9 | 39.1 | 0.0, 79.0 | 41.7 | 31.4, 52.0 |
| **Female** | | 29.6 | 24.8, 34.4 | 21.8 | 17.8, 25.8 | 34.4 | 28.7, 40.1 | 28.9 | 26.4, 31.4 |
|  | 20-29 | 16.3 | 3.0, 29.6 | 4.4 | 0.0, 9.1 | 29.6 | 19.2, 40.1 | 17.8 | 9.9, 25.8 |
|  | 30-39 | 23.4 | 12.9, 34.0 | 16.1 | 8.9, 23.4 | 31.9 | 4.3, 22.6 | 22.3 | 17.3, 27.2 |
|  | 40-49 | 21.9 | 9.9, 33.9 | 22.2 | 14.1, 30.2 | 35.8 | 24.2, 47.5 | 25.9 | 18.7, 33.1 |
|  | 50-59 | 39.2 | 25.8, 52.5 | 39.4 | 27.7, 51.0 | 36.6 | 25.1, 48.1 | 36.9 | 28.3, 45.5 |
|  | 60-69 | 33.8 | 25.5, 42.0 | 30.5 | 24.1, 37.0 | 49.6 | 39.1, 60.2 | 35.7 | 29.6, 41.8 |
|  | 70-79 | 41.3 | 30.6, 52.1 | 26.0 | 11.7, 40.4 | 28.8 | 14.8, 42.8 | 38.7 | 29.2, 48.2 |
|  | 80-89 | 27.7 | 15.4, 40.1 | 5.7 | 0.0, 12.8 | 14.3 | 0.0, 40.1 | 23.9 | 14.1, 33.8 |
| **Total** | | 34.9 | 30.8, 39.0 | 22.7 | 20.0, 25.4 | 40.5 | 37.1, 44.0 | 34.1 | 31.3, 36.8 |
| **NAFLD by CAP cut off point of 302 dB/m** | | | | | | | | | |
| **Male** | | 35.8 | 30.3, 41.3 | 19.8 | 14.6, 25.1 | 38.0 | 33.6, 42.5 | 33.9 | 29.4, 38.3 |
|  | 20-29 | 21.5 | 7.0, 36.0 | 7.6 | 3.5, 9.6 | 25.0 | 12.9, 37.1 | 20.2 | 11.4, 29.0 |
|  | 30-39 | 24.0 | 13.4, 34.7 | 15.4 | 5.1, 25.7 | 39.7 | 25.4, 54.0 | 26.8 | 19.5, 34.0 |
|  | 40-49 | 40.5 | 22.9, 58.2 | 26.1 | 11.7, 40.5 | 46.0 | 30.9, 61.2 | 39.2 | 26.2, 52.1 |
|  | 50-59 | 40.7 | 28.4, 53.0 | 33.4 | 17.4, 49.3 | 42.1 | 30.9, 53.3 | 40.8 | 31.1, 50.5 |
|  | 60-69 | 44.5 | 32.5, 56.4 | 2.3 | 15.9, 30.8 | 41.5 | 30.4, 52.5 | 40.1 | 32.6, 47.6 |
|  | 70-79 | 46.4 | 35.0, 57.9 | 25.7 | 8.4, 43.1 | 52.8 | 30.6, 75.1 | 44.0 | 34.2, 53.7 |
|  | 80-89 | 33.1 | 21.0, 45.2 | 13.6 | 0.0, 27.9 | 39.1 | 0.0, 78.9 | 33.5 | 22.7, 44.3 |
| **Female** | | 23.6 | 19.2, 28.0 | 17.5 | 13.9, 21.2 | 26.8 | 20.9, 32.6 | 23.0 | 20.7, 25.3 |
|  | 20-29 | 12.9 | 1.5, 24.2 | 4.4 | 0.0, 9.1 | 18.9 | 10.4, 27.3 | 12.9 | 6.2, 19.5 |
|  | 30-39 | 19.4 | 10.3, 28.5 | 11.3 | 6.1, 16.4 | 23.3 | 13.6, 33.0 | 17.5 | 12.8, 22.3 |
|  | 40-49 | 17.0 | 6.3, 27.7 | 15.0 | 6.4, 23.6 | 32.8 | 20.1, 45.5 | 21.2 | 13.6, 28.8 |
|  | 50-59 | 34.9 | 22.6, 47.2 | 33.8 | 24.0, 43.7 | 29.5 | 16.1, 43.0 | 32.2 | 24.3, 40.1 |
|  | 60-69 | 26.6 | 17.5, 35.8 | 23.8 | 18.5, 29.1 | 41.6 | 31.8, 51.4 | 28.9 | 22.1, 35.6 |
|  | 70-79 | 28.2 | 16.6, 39.9 | 26.0 | 11.7, 40.4 | 21.1 | 8.1, 34.2 | 26.7 | 16.9, 36.5 |
|  | 80-89 | 20.0 | 8.5, 31.6 | 2.1 | 0.0, 6.2 | 14.3 | 0.0, 40.1 | 17.3 | 7.8, 26.7 |
| **Total** | | 29.6 | 26.0, 33.2 | 18.6 | 16.0, 21.1 | 32.3 | 28.4, 36.1 | 28.3 | 26.0, 30.6 |
