# Supplementary material for: Prevalence and factors associated with NAFLD detected by vibration controlled transient elastography among US adults: Results from NHANES 2017–2018
Source: PLoS One. 2021 Jun 3;16(6):e0252164. doi: 10.1371/journal.pone.0252164 (PMC8174685; doi:10.1371/journal.pone.0252164)
Supplement: S6 Table — (DOCX) [file pone.0252164.s006.docx]

| **S6 Table**. Multivariable analysis for factors associated with NAFLD by CAP cut off point of 290 dB/m | | | | |
| --- | --- | --- | --- | --- |
| **Variables** |  | **Multivariable adjusted OR^a^** | **95%CI** |  |
| Age |  |  |  |  |
|  | 20-29 | Ref |  |  |
|  | 30-39 | 0.96 | 0.63-1.45 |  |
|  | 40-49 | 1.50 | 1.09-2.05 |  |
|  | 50-59 | 1.81 | 1.11-2.95 |  |
|  | 60-69 | 2.11 | 1.15-3.85 |  |
|  | 70-79 | 1.70 | 1.10-2.62 |  |
|  | 80-89 | 1.27 | 0.81-1.99 |  |
| Sex |  |  |  |  |
|  | Male | Ref |  |  |
|  | Female | 0.55 | 0.37-0.66 |  |
| Race |  |  |  |  |
|  | Non-Hispanic White | Ref |  |  |
|  | Non-Hispanic Black | 0.51 | 0.40-0.66 |  |
|  | Hispanics | 1.34 | 1.02-1.75 |  |
|  | Other | 1.01 | 0.75-1.37 |  |
| Body mass index^*^ | |  |  |  |
|  | Underweight (<18.5) | 1.81 | 0.20-16.44 |  |
|  | Normal (18.5 to 25) | Ref |  |  |
|  | Overweight (25–29.9) | 6.22 | 3.43-11.31 |  |
|  | Obesity (≥30) | 26.92 | 14.83-48.86 |  |
| Hyperlipidemia^*^ | |  |  |  |
|  | Yes | 2.11 | 1.48-3.02 |  |
|  | No | Ref |  |  |
| Diabetes^*^ | |  |  |  |
|  | Normal | Ref |  |  |
|  | Pre-diabetes | 1.95 | 1.37-2.79 |  |
|  | Diabetes | 3.70 | 2.26-6.06 |  |
| Metabolic Syndrome | |  |  |  |
|  | Yes | 5.14 | 4.07-6.48 |  |
|  | No | Ref |  |  |
| Hypertension^*^ | |  |  |  |
|  | Yes | 1.54 | 1.18-2.01 |  |
|  | No | Ref |  |  |
| Physical activity | |  |  |  |
|  | Inadequate | 1.37 | 0.98-1.93 |  |
|  | Adequate | Ref |  |  |
| Macronutrients | |  |  |  |
|  | Average total energy intake  (100 unit increase) | 0.87 | 0.25-2.96 |  |
|  | Carbohydrate intake | 0.99 | 0.95-1.04 |  |
|  | (10 unit increase) |  |  |  |
|  | Total fat (10 unit increase) | 1.04 | 0.90-1.20 |  |
| ^*^ Final model adjusted without metabolic syndrome | | |  |  |
| ^a^ Final model including age, sex, race physical activity, total energy intake, carbohydrate intake, total fat with either metabolic syndrome or obesity, diabetes, hypertension, hyperlipidemia. | | | | |
