# Supplementary material for: Prevalence and factors associated with NAFLD detected by vibration controlled transient elastography among US adults: Results from NHANES 2017–2018
Source: PLoS One. 2021 Jun 3;16(6):e0252164. doi: 10.1371/journal.pone.0252164 (PMC8174685; doi:10.1371/journal.pone.0252164)
Supplement: S7 Table — (DOCX) [file pone.0252164.s007.docx]

| **S7 Table**. Multivariable analysis for factors associated with NAFLD by CAP cut off point of 302 dB/m | | | |
| --- | --- | --- | --- |
| **Variables** |  | **Multivariable adjusted OR^a^** | **95%CI** |
| Age |  |  |  |
|  | 20-29 | Ref |  |
|  | 30-39 | 1.08 | 0.73-1.59 |
|  | 40-49 | 1.70 | 1.19-2.42 |
|  | 50-59 | 2.06 | 1.19-3.58 |
|  | 60-69 | 2.10 | 1.13-3.93 |
|  | 70-79 | 1.59 | 1.03-2.44 |
|  | 80-89 | 1.16 | 0.67-2.04 |
| Sex |  |  |  |
|  | Male | Ref |  |
|  | Female | 0.49 | 0.33-0.73 |
| Race |  |  |  |
|  | Non-Hispanic White | Ref |  |
|  | Non-Hispanic Black | 0.49 | 0.37-0.65 |
|  | Hispanics | 1.09 | 0.77-1.54 |
|  | Other | 0.92 | 0.61-1.39 |
| Body mass index^*^ | |  |  |
|  | Underweight (<18.5) | 2.84 | 0.29-28.23 |
|  | Normal (18.5 to 25) | Ref |  |
|  | Overweight (25–29.9) | 6.15 | 3.17-11.92 |
|  | Obesity (≥30) | 29.47 | 16.14-53.81 |
| Hyperlipidemia^*^ | |  |  |
|  | Yes | 1.62 | 1.11-2.38 |
|  | No | Ref |  |
| Diabetes^*^ | |  |  |
|  | Normal | Ref |  |
|  | Pre-diabetes | 1.98 | 1.39-2.81 |
|  | Diabetes | 4.33 | 2.95-6.36 |
| Metabolic Syndrome | |  |  |
|  | Yes | 5.76 | 4.32-7.70 |
|  | No | Ref |  |
| Hypertension^*^ | |  |  |
|  | Yes | 1.71 | 1.26-2.32 |
|  | No | Ref |  |
| Physical activity | |  |  |
|  | Inadequate | 1.52 | 1.02-2.27 |
|  | Adequate | Ref |  |
| Macronutrients | |  |  |
|  | Average total energy intake  (100 unit increase) | 1.12 | 0.25-5.15 |
|  | Carbohydrate intake | 0.98 | 0.92-1.04 |
|  | (10 unit increase) |  |  |
|  | Total fat (10 unit increase) | 1.00 | 0.85-1.19 |
| ^*^ Final model adjusted without metabolic syndrome | | |  |
| ^a^ Final model including age, sex, race physical activity, total energy intake, carbohydrate intake, total fat with either metabolic syndrome or obesity, diabetes, hypertension, hyperlipidemia. | | | |
